# Shifting COVID-19 Vaccine Intentions in New Zealand: Next Steps in the Vaccination Campaign

**DOI:** 10.1016/j.lanwpc.2021.100278

**Published:** 2021-09-17

**Authors:** Jagadish Thaker, Brian Floyd

**Affiliations:** a School of Communication, Journalism & Marketing, Massey University, Wellington, New Zealand; b Independent Researcher

**Keywords:** COVID-19 vaccine, vaccine hesitancy, vaccine intentions, pandemic, New Zealand, Ethnicity and health outcomes

Few studies inform us about the New Zealand public intentions to get a COVID-19 vaccine, even as the vaccine rollout has been slow—a pattern similar to Australia, unlike several other developed countries [1]. Such low vaccination rates pose an increasing threat from the new COVID-19 variants to New Zealand, which has been able to evade the pandemic so far [2]. Given the brittle health care system in New Zealand—with second-fewest intensive care beds per capita among developed countries—health experts say it is important to vaccinate a very large proportion, about 80-90%, of the population to safely open the borders [2].

A recent study by Prickett and colleagues [3] in the *Lancet Regional Health – Wester Pacific* found that while a majority of New Zealanders (71%) say they are likely to take a COVID-19 vaccine, a significant minority were unsure (15%) or unlikely (14%) to get a vaccine. Young and less educated were more likely to decline a vaccine. Moreover, women were more likely to be unsure than men who were more certain about getting a vaccine or not. However, they did not find ethnicity as significantly associated with COVID-19 vaccine hesitancy in a multivariate model, suggesting that “public health focus on perceived hesitancy in the Māori and Pacific populations—subgroups that are particularly at-risk of COVID-19 infection and morbidity—may be misplaced” (p. 2).

Supplementing their findings, only age and education were significantly associated with COVID-19 vaccine intentions in online national surveys conducted in March 2021 (Wave 1=1083) and May 2021 (Wave 2=650). Similarly, ethnicity was not significantly associated with vaccine intentions in the presence of other socio-demographic variables; neither was gender or income.

In longitudinal online nationally representative surveys conducted between March^1^ and May 2021 (*N*=650), there is a six-percentage point increase among the New Zealand respondents who will ‘definitely’ take COVID-19 vaccine to protect themselves from 61% in March 2021 to 67% in May 2021 (67%). However, a quarter remained unsure (26%), and another 8% were sceptical. The vaccine uptake is at its highest in over a year across multiple public opinion surveys [4,5]. This finding is consistent with the Ministry of Health surveys that showed an uptick in May [4].

A large majority (92%) of those who had made up their mind to ‘definitely’ get a vaccine in March continued to do so in May. A small minority became more hesitant (7%), while 1% became more sceptic. The biggest shift was among those who were ‘Unsure, but leaning towards YES’ in March to ‘definitely’ in May (42%), even as about half continued to be unsure (47%) and just over 11% became more hesitant or sceptic. The second largest shift was among those who would ‘definitely’ not get a vaccine in March: while a majority still continued to say so (64%), 21% shifted to ‘Unsure, but leaning towards NO,’ 4% to ‘Unsure, but leaning towards YES’, and 11% to ‘definitely’ getting a vaccine (See Figure 1).

**Figure 1:**
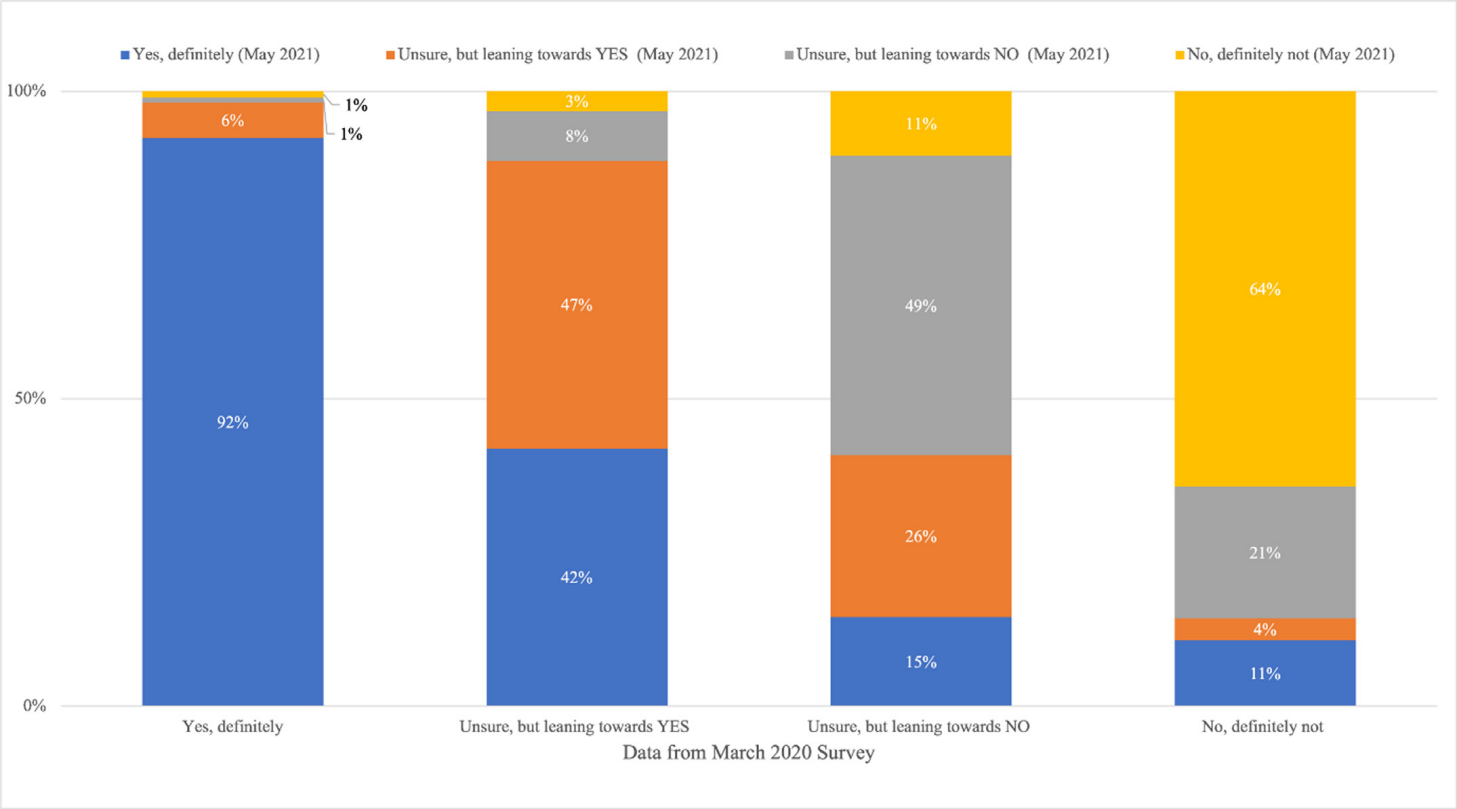
Shifting COVID-19 Vaccine Intentions in New Zealand between March and May 2021

The uptick in COVID-19 vaccine intentions, compared to those who did not change their intentions between March and May 2021, was primarily due to uptick among Māori and Asians. Māori respondents were 2 times (Odds Ratio [OR] = 2.28, [1.22, 4.26]) more likely to become more enthusiastic about COVID-19 vaccination in preceding three months compared to European New Zealanders, holding other socio-demographic variables constant. Asians (Odds Ratio [OR] = 2.19, [1.15, 3.91]) were also twice as likely as European New Zealanders to become more motivated to get a COVID-19 vaccine in three months. Gender, age, education, gender, and income were not associated with change in COVID-19 vaccine intentions. In other words,

At the same time, there were no significant association between socio-demographic factors and a decline in intentions compared to those who did not change their intention, except for a minor increase in vaccine hesitancy among younger aged groups. Consistent with findings from other Ministry of Health surveys [4], the COVID-19 vaccination campaign should seek ways to understand and engage the young and less educated groups.

The uptick among the ethnic minorities of Māori and Asians is good news. It implies the success of both community-led COVID-19 protective measures as well as government outreach [3,6]. Māori and Pasifika health advocates leading the fight against vaccine misinformation [7], promoting vaccination through pop-up clinics, and organising vaccination centres at and during community events has helped close the vaccination gap between Māori and non-Māori [8].

Several studies show that ethnic minorities have lower intentions to get a COVID-19 vaccine, in part due to historic inequalities, differential access to health care, among others [9]. Even prior surveys in New Zealand show vaccination intention is generally low among minorities [4,5], even as their risk of hospitalization and death is higher than others [10]. Continuing the government's focus to engage with at-risk groups will help build trust to resolve other persisting public health issues (Table 1).

**Table 1:** Longitudinal analysis of change in COVID-19 vaccine intentions between March and May 2021 in New Zealand

	Less hesitant to get a COVID-19 vaccinevs.no change in intention						More hesitant to get a COVID-19 vaccinevs.no change in intention					
	*B*	*S.E.*	*Sig.*	*Exp(B)*	*95% C.I. for EXP(B)*		*B*	*S.E.*	*Sig.*	*Exp(B)*	*95% C.I. for EXP(B)*	
Constant	-2.05	0.50	0.00	0.13			-3.73	0.78	0.00	0.02		
**Gender**												
Male	0.06	0.23	0.81	1.06	0.67	1.67	-0.09	0.31	0.77	0.91	0.50	1.68
**Age**												
18-25	-0.11	0.51	0.83	0.90	0.33	2.42	1.37	0.65	0.04	3.94	1.10	14.08
26-35	0.38	0.39	0.32	1.46	0.69	3.12	0.85	0.60	0.15	2.35	0.73	7.57
36-45	0.49	0.40	0.22	1.63	0.74	3.59	1.57	0.58	0.01	4.82	1.54	15.04
46-55	-0.26	0.39	0.50	0.77	0.36	1.65	0.80	0.56	0.16	2.22	0.74	6.66
56-65	0.41	0.36	0.25	1.51	0.75	3.06	0.82	0.57	0.15	2.27	0.74	6.96
**Education**												
No qualification	0.74	0.46	0.11	2.09	0.85	5.10	0.41	0.64	0.52	1.51	0.43	5.25
School qualification	0.32	0.32	0.33	1.37	0.73	2.58	0.43	0.42	0.30	1.54	0.68	3.48
Tertiary diplomas/Certificates	0.34	0.30	0.25	1.41	0.78	2.54	0.26	0.39	0.51	1.30	0.60	2.81
**Ethnicity**												
Māori	0.82	0.32	0.01	2.28	1.22	4.26	-0.05	0.49	0.91	0.95	0.37	2.46
Pasifika	-0.05	0.65	0.94	0.96	0.27	3.41	-0.36	0.80	0.65	0.70	0.15	3.31
Asian or Another	0.75	0.31	0.02	2.12	1.15	3.91	-0.36	0.46	0.43	0.70	0.29	1.71
**Income**												
Less than $19,999	0.01	0.43	0.98	1.01	0.44	2.32	-1.06	0.81	0.19	0.35	0.07	1.70
$20,000 to $39,999	-0.36	0.43	0.41	0.70	0.30	1.63	0.40	0.65	0.54	1.49	0.42	5.36
$40,000 to $59,999	-0.20	0.41	0.63	0.82	0.36	1.85	0.52	0.62	0.40	1.69	0.50	5.68
$60,000 to $79,999	-0.63	0.43	0.14	0.53	0.23	1.24	0.70	0.60	0.25	2.01	0.62	6.52
$80,000 to $99,999	-1.19	0.62	0.05	0.30	0.09	1.02	0.62	0.68	0.37	1.85	0.49	7.09
Pseudo *R*^2^	0.07						0.07					

*Note: N*=650; Female compared to male. Those 66 years and above were reference category for age. Bachelors or higher degree was the reference category for education. Ethnicity dummy coded with reference to European-New Zealanders. Individuals with $100,000 or more annual income was the reference category for income.

It is also important to highlight that there is a vaccination uptake gap among Māori and Pacific people compared to the general population and the gap is slow to narrow down [2]. Understanding the shifting public intentions to get a COVID-19 vaccine will better help increase vaccination uptake, particularly important as almost all COVID-19 deaths are now among the unvaccinated in other countries [11]. On the one hand, health care system should be more embedded with at-risk communities. On the other, we need more participation from local groups—from community and local councils, business groups, unions, and others—encouraging vaccine uptake among their group members.

## Declaration of Competing Interest

The authors declare no conflict of interest.

## Figures and Tables

**Table A1:** Sample Demographics

	(Full sample, March 2021)		(Panel, May 2021)	
Total	*N* = 1083	%	*n* = 650	%
**Gender**				
Female	552	51	324	50
Male	531	49	326	50
**Age**				
18-25	155	14	53	8
26-35	215	20	115	18
36-45	174	16	81	13
46-55	193	18	138	21
56-65	162	15	110	17
66 and above	184	17	153	24
**Education**				
No qualification	87	8	45	7
School qualification	286	26	177	27
Tertiary diplomas/Certificates	335	31	199	31
Bachelor's degree or higher	375	35	229	35
**Ethnicity**				
European New Zealander	689	64	457	70
Māori	175	16	69	11
Pasifika	43	4	25	4
Asian or Another	176	16	99	15
**Annual income**				
Less than $19,999	208	19	121	19
$20,000 to $39,999	261	24	164	25
$40,000 to $59,999	197	18	124	19
$60,000 to $79,999	184	17	110	17
$80,000 to $99,999	94	9	63	10
$100,000 to $119,999	66	6	30	5
$120,000 or above	66	6	34	5
**Intentions to get a COVID-19 vaccine**				
Yes Definitely	394	61	433	67
Unsure but leaning towards YES	124	19	103	16
Unsure but leaning towards NO	76	12	62	9
No Definitely not	56	9	52	8

## Appendix

Table A1.

## Footnotes

1 Full Wave 1 (*N*=1083) (compared to longitudinal Wave 1): 56% ‘Yes, definitely’ (61%), 22% ‘Unsure, but leaning towards YES’ (19%), 12% ‘Unsure, but leaning towards NO’ (12%), 10% ‘No, definitely not’ (9%). The reinterview response rate was 60% between March and May 2021. Compared to the full wave 1 sample, the longitudinal wave 1 sample had slightly higher vaccine acceptance rate (61% as mentioned above) but similar hesitancy and refusal rates.
